# Embryo and Fetal Toxic Effects of the Hydroethanol Extract of *Urtica simensis* Hochst. Ex. A. Rich Leaves in Pregnant Rats

**DOI:** 10.1155/2024/9986648

**Published:** 2024-11-08

**Authors:** Bickes Wube, Kaleab Asres, Samuel Woldekidan, Abiy Abebe, Yonas Girma, Girma Seyoum

**Affiliations:** ^1^Department of Anatomy, College of Health Sciences, Addis Ababa University, Addis Ababa, Ethiopia; ^2^Department of Pharmaceutical Chemistry and Pharmacognosy, College of Health Sciences, Addis Ababa University, Addis Ababa, Ethiopia; ^3^Traditional and Modern Medicine Research Directorate, Ethiopian Public Health Institute, Addis Ababa, Ethiopia; ^4^Department of Pathology, College of Health Sciences, Addis Ababa University, Addis Ababa, Ethiopia

**Keywords:** embryo, fetus, toxicity, *Urtica simensis*, Wistar albino rat

## Abstract

**Introduction:** *Urtica simensis* has been used to treat various diseases such as malaria, hypertension, diabetes, gonorrhea, gastritis, body swelling, and wound infections. However, the safety of consuming *U. simensis* leaves during pregnancy has not been evaluated yet. Therefore, this experimental study was conducted to evaluate the toxic effects of *U. simensis* leaf extract on the prenatal development of embryos and fetuses in pregnant rats.

**Methods:** Fifty pregnant Wistar albino rats were randomly assigned to five groups of 10 gravid rats for each experiment. Groups I–III were given 70% ethanol leaf extract of *U. simensis* at doses of 250, 500, and 1000 mg/kg daily from 6^th^ to 12^th^ days of gestation. Groups IV–V were kept as pair-fed and ad libitum controls. The developing embryos and fetuses were retrieved on 12 days and 20 days of gestation, respectively. Embryos were evaluated for growth and developmental delays. Fetuses were also assessed for growth retardation and external and visceral anomalies.

**Results:** In the embryonic experiment, somite numbers (*p*=0.001) and morphological scores (*p*=0.029) were significantly decreased in pregnant rats given 1000 mg/kg of *U. simensis* leaf extract. Embryonic developments of the caudal neural tube (CNT) (*p*=0.001), otic system (*p*=0.025), olfactory system (*p*=0.013), and limb buds (*p*=0.026) were significantly delayed in pregnant rats given 1000 mg/kg of extract. Oral administration of 500 mg/kg of *U. simensis* leaf extract also caused significant developmental delays in the CNT (*p*=0.021) and olfactory system (*p*=0.032). In the fetal experiment, fetal resorption (*p*=0.015) was significantly increased whereas crown rump length (*p*=0.012) and fetal weight (*p*=0.019) were significantly decreased in pregnant rats given 1000 mg/kg of *U. simensis* leaf extract.

**Conclusions:** The embryotoxic effects of *U. simensis* leaf extract were evidenced by significant developmental delays. The fetal toxic effects of *U. simensis* leaf extract were also shown by significant decreases in fetal growth indices. Therefore, pregnant women should be well informed of the possible toxic effects of consuming *U. simensis* leaf during pregnancy.

## 1. Introduction

Traditional medicines and therapeutic provisions are increasingly popular around the world [1]. More than 85% of world population uses medicinal herbs to prevent and treat diseases [2]. Indigenous medicinal herbs are often preferable, accessible and cheap in developing nations [3]. In Africa, herbal medicines are essential for maintaining health and wellbeing of the population. However, the main challenges that hinder its effective use are lack of quality control and safety measures [4]. The use of plant medicine among pregnant mothers has substantially increased throughout the world, especially in sub-Saharan Africa [5]. The use of plant medicine during pregnancy varies based on geography, race, cultural norms, and economic status [6]. Utilization of herbal remedies lacking clear pharmacological activities may pose risks to pregnant women [7]. A multinational study assessed the safety of 126 herbal remedies frequently utilized by pregnant women [8]. However, only 28 of them (22.2%) have been identified as favorable during pregnancy. In Africa, the mean herbal medication among pregnant women varied from 32% to 45% [9]. Recent studies done in sub-Saharan Africa revealed that the use of herbal remedies during pregnancy ranged from 2% in northern Ethiopia [10] to 100% in eastern Kenya [11]. In Ethiopia, 47.77% of the mothers having prenatal care used herbal medication during pregnancy [12], ranging from 10.9% [13] to 73.1% [14]. Another recent study revealed that the use of Prunus Dulcis was related to preterm birth, oral raspberry leaf with caesarean delivery, and excessive consumption of liquorice with early preterm birth [15]. Medical herbs are distinguished by different plant species. For example, the genus *Urtica* includes more than 80 species found across the world [16]. *Urtica simensis* Hochst. ex. A. Rich belongs to the genus *Urtica* and the family *Urticaceae* [16]. It is a native medicinal plant in Ethiopia and locally known as samma in Amharic [17], ameie in Tigrigna [18], sanamik in Halaba [19], and dobii or gurgubbee in Oromifaa [20]. The phytochemical screening of *U. simensis* leaf revealed phenols, flavonoids, oxalate, terpenoids, alkaloids, and tannins [21]. The plant also contains aromatic hydrocarbons such as p-xylene, o-cymene, and p-cymene [22]. Proximate analysis of *U. simensis* leaf also showed the presence of ash carbohydrate, crude fiber, protein, and fat [23]. In Ethiopian folk medicine, the *U. simensis* leaves were used for treatment of gastritis [24], stomach ulcers [25], intestinal parasites [26], sexually transmitted diseases [17], heart failure [27], bleeding wounds [28], diabetics [21], hemorrhoids [29], nyctalopia [30], and gonorrhea [31]. The efficacies of *U. simensis* leaves were also proven for antidiabetic [32], antiulcer [33], wound healing [34], antimicrobial [35], cardioprotective [36], antiproliferative [22], and antioxidant [37] effects. To date, no teratogenic studies have been reported on this plant. Teratogenic studies are the third most serious issue with respect to drug formulations after cardiovascular and liver toxicity studies [38]. Evaluation of the developmental toxicity of any medication has become mandatory before human use [39]. Prenatal developmental toxicity studies of medicinal herbs during pregnancy are essential and relevant. Therefore, this experimental study was conducted to evaluate the toxic effects of the *U. simensis* leaf extract on the prenatal development of embryos and fetuses in pregnant rats.

## 2. Materials and Methods

### 2.1. Plant Collection and Authentication

Fresh *U. simensis* leaves were collected in Gozamen woreda, 310 km northwest of Addis Ababa, the capital city of Ethiopia. The leaves were randomly collected from bottoms to tips according to their stalk positions using disinfected safety gloves. Samples of collected leaves were placed in polybag and submitted to the Addis Ababa University (AAU) National Herbarium for identification and authentication. A voucher sample (bw001) was put in the National Herbarium for future reference.

### 2.2. Proximate Analysis

The moisture, ash, crude protein, crude fat, crude fiber, and carbohydrate contents of *U. simensis* leaves were determined using the Association of Official Analytical Chemists (AOACs) standard procedures [40]. Air dried leaves of *U. simensis* (10 g) were placed in 100°C oven to determine the moisture content. The weight of the dish before drying (W1) and after drying (W2) was weighed and their difference (W2 minus W1) was determined. The moisture content was calculated by subtracting the weight difference of dish from weight of the sample (10 g), divided by sample weight and multiplied by 100. The same amount of *U. simensis* leaf was heated in muffle furnace at 600°C to determine total ash content. Then, the ash content was determined by subtracting the weight of the crucible from weight of the crucible with ash, divided by 10 g and multiplied by 100. The crude fat content of 10 g of air-dried leaves of *U. simensis* was also determined using the Soxhlet apparatus with hexane extraction. It was calculated by subtracting weight of the flask from weight of flask with fat, dividing by 10 g and multiplying by 100. The total crude fiber content of 10 g sample was also determined by successively boiling in 0.313 M H2SO4 acid and 0.313 M KOH base solutions, rinsed in boiled water and acetone, dried, weighed and incinerated in the furnace at 550°C. The crude fiber content was determined by subtracting the weight of the crucible with ash from the weight of crucible with fiber, dividing by 10 g and multiplying by 100. The total crude protein of the sample was estimated using the Kjeldahl digestion and distillation method with conversion factor of 6.25 based on the total nitrogen content and multiplied by the dilution factor [41]. Finally, the subtraction method was used to determine the total carbohydrate content. It was computed by subtracting the total dry weight of the sample from crude protein, crude fiber, total ash, and crude fat [42].

### 2.3. Phytochemical Screening

Qualitative phytochemical screening tests for the 70% ethanol extract of *U. simensis* leaves were carried out to detect the presence of phenols, flavonoids, tannins, terpenoids, saponins, steroids, alkaloids, and anthraquinones according to the methods described by Khan et al. [43].

### 2.4. Extraction

The collected *U. simensis* leaves were cleaned using distilled water, air dried, and manually crushed into pieces before being coarsely pulverized with an electric grinder. The coarsely milled leaves were weighted using an electronic balance (METTLER TOLEDO, Switzerland). These roughly ground leaves were mixed with 70% ethanol in powder to solvent ratio of 1–10 (w/v) and constantly oscillated for 24 h using an orbital shaker (Bibby scientific limited stone, UK). Whatman no. 1 filter paper (Maid stone, UK) was employed to filter the agitated mixture. After filtration, the residue underwent double maceration process over 2 days with 70% ethanol. The obtained filtrate was evaporated using a rotatory evaporator (Büchi R-200, Switzerland) at controlled temperature not exceeding 40°C. The residual aqueous solution was desiccated in 40°C water bath for fortnight. The desiccated extract was stored in the refrigerator until it was used [44].

### 2.5. Laboratory Animals

A total of 75 Wistar albino rats (25 males and 50 nulliparous females) weighted 220–250 g and aged 10–12 weeks were selected for each experiment. These rats were sourced from the animal breeding unit at the Ethiopian Public Health Institute (EPHI). They were housed in standard unstained steel cages under normal light-dark cycles and kept at room temperature (23 ± 3°C) with relative humidity of 50 ± 10%. Rats were given conventional laboratory diet and drinking water daily. After 1 week of acclimatization, rats were mated overnight by paring one male rat with two female rats. The next morning, female rats were examined for copulatory plug and vaginal smears were taken for microscopic examination of sperm. The first day observation of sperm in vaginal swab was taken as day one of pregnancy [45].

### 2.6. Experimental Designs

An acute oral toxicity assessment of *U. simensis* leaf extract was conducted to determine the median lethal dose (LD_50_) following OECD guideline 425 [46]. Based on the up and down oral toxicity test, LD_50_ of *U. simensis* leaf extract was greater than 5000 mg/kg animal body weight. The experimental doses were determined with two to four fold intervals based on the LD_50_ dose. The highest dose was set as 1000 mg/kg which was one fifth of the LD50 dose. The middle dose was 500 mg/kg, half of highest dose, and lowest dose was 250 mg/kg, one fourth of highest dose. Then, five groups of 10 gravid rats were randomly assigned using OECD guidelines for developmental toxicity studies [47, 48]. Groups I–III were treatment groups whereas group IV and group V were pair-fed and ad libitum controls, respectively. The treatment groups were administered *U. simensis* leaf extract at doses of 250, 500, and 1000 mg/kg animal body weight. The pair-fed group received only distilled water at 1 mL/100 g body weight. The ad libitum control was given food and water freely. The extract was administered through gavage from 6^th^ to 12^th^ days of gestation as it was critical period of embryogenesis and organogenesis in rats [49]. The daily dietary intake of each group was measured. The weight of each rat was also taken at first confirmation of pregnancy, day 6, day 12, and day 20 of gestation [50].

### 2.7. Embryonic Experiment

On the 12^th^ day of gestation at noon, gravid rats were given pentobarbital 150 mg/kg through intraperitoneal injection to induce unconsciousness [51]. Afterward, the rats were positioned supine on the operating table. Then, an abdominal incision was done to reveal abdominal cavities. Pins were used to fix skin flaps and abdominal muscles on both sides. The uterine horns were retrieved and placed in Hank's solution. An incision was made along antimesometrial boundary of uterine horns to reveal the developing embryos [52, 53]. The embryonic membranes were meticulously separated to expose the visceral yolk sac and the circulation was observed through dissecting microscope. The embryonic development of each system was assessed using the Brown and Fabro criteria [54] later adopted by Seyoum and Persaud [55]. The crown rump length and somites of each embryo were also measured and counted.

### 2.8. Fetal Experiment

On the 20^th^ day of gestation at noon, pregnant rats were given pentobarbital 150 mg/kg through intraperitoneal injection to induce unconsciousness [51]. The techniques used to recover the fetuses were similar to the embryonic experiment. Uterine horns were fully exposed and examined. The yellowish nodules of metrial glands along mesometrial border of uterine horns were assessed to count implantation sites. The resorptions were identified by metrial glands that were not held by alive or deceased fetuses. Gently pressing on the fetus revealed whether it was alive or dead.

#### 2.8.1. External Examination

Fetuses were retrieved and their placentas were detached. The crown rump length was measured from top of the head to the buttocks. The fetal and placental weights were recorded. Each fetus was examined for craniofacial development abnormalities (exencephaly, anencephaly, microphthalmia, and anophthalmia), limb development abnormalities (syndactyly, adactyly, and polydactyly), vertebral column malformations (neural tube defect, kyphosis, and scoliosis), tail development disorders (lost tail), and external genitalia abnormalities. After external examination, two to three fetuses from each rat were immersed in 95% ethanol for 1 week. These alcohol preserved fetuses were eviscerated through laparotomy and subjected to skeletal staining using the revised techniques of Rigueur and Lyons [56]. The remaining fetuses were fixed in Bouin's solution for visceral examination [57].

#### 2.8.2. Visceral Examination

Bouin's solution-fixed fetuses were serially sectioned for visceral examination. Using dissecting microscope and the revised Wilson technique [23], coronal section of the head and transverse section of the rest of the body were performed to examine for readily apparent visceral abnormalities in the head, neck, thorax, and abdominopelvic regions.

#### 2.8.3. Skeletal Examination

A skeletal scoring chart developed by Nash and Persaud [58] was used to evaluate skeletal development. The alizarin stained skeletons were examined through dissecting microscope. Evaluation and quantification of ossification centers were conducted. The degree of ossification of the sternebrae, metacarpal, metatarsal, and sacrococcygeal bones were taken as key markers of skeletal development [59]. Furthermore, ossifications of the skull, hyoid, sternum, ribs, vertebrae, and limb bones were also scrutinized.

### 2.9. Statistical Analysis

Data were cleaned, coded and entered in EpiData Version 3.1. Then, it was exported to Statistical Package for Social Science (SPSS) Version 25 for analysis. The Shapiro–Wilk and Levene's tests were used to assess normality of data and homogeneity of variance, respectively. One-way analysis of variance (ANOVA) with Tukey's post hoc test was done to compare within and between treatment and control groups. Mixed-model ANOVA was used to analyze dietary intake and weight changes within and between groups over time. Data were reported using mean and standard deviation. Findings were considered statistically significant if the *p* value was less than 0.05.

## 3. Results

### 3.1. Proximate Composition and Secondary Metabolites

The proximate analysis of *U. simensis* leaf revealed 7.4% moisture, 28% total ash, 31% crude protein, 3.2% crude fat, 7% crude fiber, and 30.8% carbohydrate. Tannins and phenols were detected in ethanol, hexane, and chloroform extracts of *U. simensis* leaf but not in the aqueous extract. Moderate amounts of steroids were present in all solvent extracts. Terpenoids were only present in the ethanol extract of *U. simensis* leaf. High amounts of alkaloids and phenols were detected in the ethanol extract, which was relatively rich in phytochemicals. However, anthraquinones and glycosides were not found in all extracts (Table 1).

### 3.2. Dietary Intake and Weight Gain

There was no significant difference in daily dietary intake of pregnant rats given *U. simensis* leaf extract as compared with pair-fed and ad libitum control groups. In addition, pregnant rats administered *U. simensis* leaf extract revealed dose-dependent decrease in weight gain compared with the two control groups. However, this difference was not statistically significant (Table 2).

### 3.3. Embryo Outcomes

#### 3.3.1. Embryonic Growth Effects

The embryonic growth indices were decreased in pregnant rats given *U. simensis* leaf extract compared with the pair-fed and ad libitum control groups. Pregnant rats given 1000 mg/kg of *U. simensis* leaf extract exhibited significant decrease in somite number per litter as compared to pair-fed and ad libitum control groups. In addition, pregnant rats administered 1000 mg/kg of *U. simensis* leaf extract showed significant reduction in morphological score per litter in comparison with the control groups (Table 3).

#### 3.3.2. Embryo Developmental Effects

**Figure 1:**
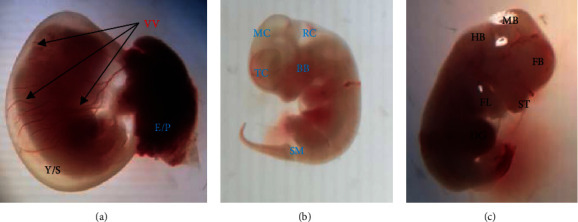
Examination of 12-days-old rat embryo through the dissecting microscope: (a) vitelline vasculature (VV), intact yolk sac (Y/S), and embryonic placenta (E/P); (b) telencephalon (TC), mesencephalon (MC), rhombencephalon (RC), brachial bars (BBs), and somites (SM); and (c) fore brain (FB), mid brain (MB), hind brain (HB), stomodeum (ST), fore limb bud (FL), and developing gut (DG).

The embryonic developments of the circulatory system, neurological system and musculoskeletal and craniofacial regions were assessed using morphological endpoints (Figure 1). Pregnant rats given 1000 mg/kg of *U. simensis* leaf extract revealed significant embryonic developmental delays of the caudal neural tube, otic system and olfactory system compared with the pair-fed and ad libitum control groups. In addition, pregnant rats administered 500 mg/kg of *U. simensis* leaf extract showed significantly low developmental scores of caudal neural tube and olfactory system as compared with control groups. Furthermore, the embryo developmental scores of forelimb and hindlimb significantly decreased in pregnant dams administered 1000 mg/kg of *U. simensis* leaf extract as compared with pair-fed and ad libitum control groups (Table 4).

### 3.4. Fetal Outcomes

**Figure 2:**
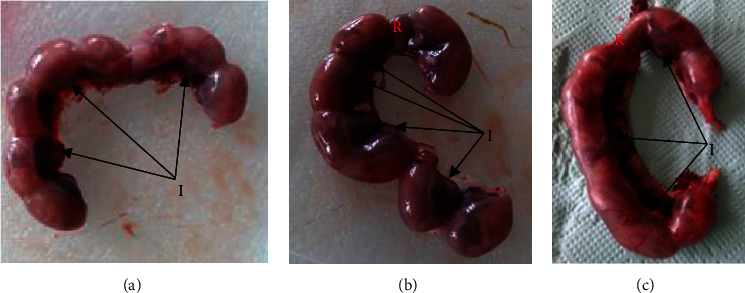
Implantation (I) and resorption (R): (a) ad libitum control, (b) 500 mg/kg, and (c) 1000 mg/kg of *Urtica simensis* leaf extract treatment groups.

Pregnant rats treated with *U. simensis* leaf extract had dose-dependent increase in fetal resorption as compared with the pair-fed and ad libitum control groups. Pregnant rats given 1000 mg/kg of *U. simensis* leaf extract revealed significant increase in fetal resorption as compared with pair-fed and ad libitum control groups. However, there was no significant difference in implantation sites per dam between treatment and control groups. No dead fetus was retrieved in all experimental groups (Table 5 and Figure 2).

#### 3.4.1. Fetal Growth Effects

Pregnant rats exposed to the *U. simensis* leaf extract showed dose-dependent reduction in fetal growth indices compared with pair-fed and ad libitum pregnant rats. Pregnant rats given 1000 mg/kg of *U. simensis* leaf extract revealed significantly reduced fetal weight compared with pair-fed and ad libitum control groups. In addition, pregnant rats given 1000 mg/kg of *U. simensis* leaf extract showed significant decrease in fetal crown rump length compared with pair-fed and ad libitum control groups. The mean placental weight in pregnant rats administered 1000 mg/kg of *U. simensis* leaf extract was low as compared with the pair-fed and ad libitum controls but not statistically significant (Table 6).

#### 3.4.2. External and Visceral Anomalies

**Figure 3:**
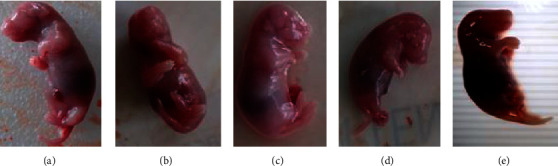
External examination of 20-days-old rat fetuses: ad libitum control group (a), pair-fed control group (b), 250 mg/kg (c), 500 mg/kg (d), and 1000 mg/kg (e) of *Urtica simensis* leaf extract treated groups.

**Figure 4:**
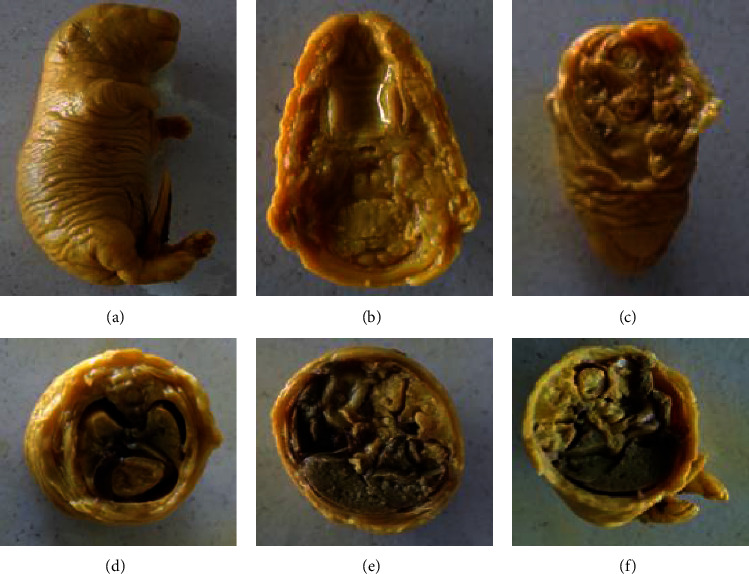
Visceral examination of Bouin's solution-fixed 20-days-old rat fetus: (a) unsectioned fetus, (b) coronal section of normal palate and brain tissue, (c) transverse section with normal viscera of neck, (d) transverse section made through thorax with intact diaphragm, (e) transverse section made through the abdomen showed normal visceral organs, and (f) transverse section through the abdominopelvic region with normal visceral organs.

The explanted fetuses were examined for external anomalies from cranial to caudal as part of treatment-related developmental anomalies such as limb deformities, vanishing tails, craniofacial malformations, vertebral column discrepancies, and apparent genital defects (Figure 3). However, there were no visible external anomalies in all experimental groups (Table 7). In addition, Bouin's solution fixed fetuses were sequentially sectioned at the head, neck, chest, abdomen, and abdominopelvic regions for visceral examination (Figure 4). These serial sections were carefully inspected through dissecting microscope for any visceral anomalies. The presence of cleft palate, hydrocephalus, eye-related anomalies, and abnormalities in thyroid gland and trachea was assessed in the head and neck regions. In addition, diaphragmatic hernia, agenesis of abdominal viscera, and external genitalia were also assessed. Yet, no visible visceral abnormalities were observed through dissecting microscope (Table 8).

#### 3.4.3. Skeletal Malformations and Ossification Delays

**Figure 5:**
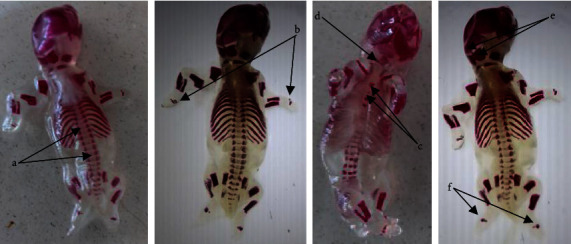
Alizarin-stained 20-days-old rat fetuses revealed different bone ossification centers: vertebrae (a), metacarpals (b), sternum (c), hyoid (d), parietal and occipital (e), and metatarsals (f).

This in vivo experimental study assessed the developments of axial and appendicular skeletons, but neither treatment nor control groups showed any notable skeletal deformities (Figure 5). However, the *U. simensis* leaf extract-treated group revealed relatively increased ossification delays in the sternum and caudal vertebrae as compared with control groups but not statistically significant (Table 9). In addition, the *U. simensis* leaf extract-treated groups revealed dose-dependent increase in ossification delays of extremity bones as compared with the pair-fed and ad libitum control groups (Table 10). However, these developmental delays were not statistically significant as compared with either of the two control groups.

## 4. Discussion

In the current study, the embryonic and fetal toxic effects of the hydroethanol extract of *U. simensis* leaves were evaluated in pregnant Wistar albino rats. The extract of *U. simensis* leaf was administered between 6 and 12 days of pregnancy as it was critical stage for growth and development [47]. The crown rump length, somites, and morphological scores were key indicators of embryonic growth [60]. Morphological scores were used to estimate embryonic development and had predictable relationship with embryonic age [54]. No deaths or noticeable behavioral changes were observed in the treatment or control groups during the entire study. The dietary intake and weight gain of pregnant rats given 1000 mg/kg of *U. simensis* leaf extract were decreased compared with pair-fed and ad libitum control groups; however, the differences were not statistically significant. This was in line with previous findings on the cardioprotective and antidiabetic properties of the *U. simensis* leaf extract, which revealed experimental animals receiving the plant extract lost weight [32, 36]. The substantial reduction in weight gain could be attributed to the presence of polyphenols in *U. simensis* leaves, which showed weight loss effect as reported by Farhat, Drummond, and Al‐Dujaili [61]. Toxicity or reaction to treatments would be other plausible explanation for weight gain reduction in the pregnant dams [62, 63]. Pregnant rats given 1000 mg/kg of *U. simensis* leaf extract revealed significant decreases in somite numbers and morphological scores compared with pair-fed and ad libitum control groups. Due to the use of crude extract, it was challenging to differentiate which bioactive ingredient was responsible for the reduction of embryonic growth parameters. However, it might be attributed to the presence of naphthalene, eugenol, or organosulfur compounds in *U. simensis* leaves as reported by a previous study [64], which deter cell cycle and promote apoptosis [65–67]. The dose-dependent decrease in the embryonic crown rump length might be due to the presence of alkaloids and other compounds such as linalool in *U. simensis* leaves which have been reported to halt growth and development [64, 68, 69]. In the present study, significant embryonic developmental delays of caudal neural tube, otic system, olfactory system, forelimb, and hindlimb were shown in pregnant rats given 1000 mg/kg of *U. simensis* leaf extract. Significant developmental lags of caudal neural tube and olfactory system were also noticed in pregnant rats administered 500 mg/kg of *U. simensis* leaf. These embryonic developmental delays could be attributed to the presence of tannins and alkaloids in the *U. simensis* leaf extract which are known to restrain growth and development through interfering transmission of cholinergic neurons [70, 71]. In the fetal experiment of the current study, external morphological evaluation of fetuses revealed no discernible treatment-related anatomical deformities in the head, neck, thoracic, and abdominopelvic visceral organs. However, crown rump length and fetal and placental weights were reduced in a dose-dependent manner in pregnant rats given the *U. simensis* leaf extract compared with pair-fed and ad libitum control groups. The reduction of these fetal biometrics could be due to the presence of some phytochemical components of *U. simensis* leaf, especially 1,8-cineole and eugenol which induce apoptotic cell death [22, 72]. It might also be attributed to other components of *U. simensis* leaves such as phenolic substances that can reach the placenta and possibly impede placental steroidogenesis [73]. Furthermore, the significant increase of fetal resorption in pregnant rats given 1000 mg/kg dose could be attributed to the apoptosis role of phytochemicals present in the *U. simensis* leaf [64]. The aromatic hydrocarbons in the *U. simensis* leaf [22] might also have role in resorption due to their intermediary effect on infertility [74]. In the present study, the ossification of sternum, ribs, vertebrae, hyoid, metacarpal, metatarsal, forelimb, and hindlimb bones did not significantly differ between treatment and control groups. However, ossification centers in the *U. simensis* leaf extract-treated groups were lower than those in the control groups. These nonsignificant differences suggested that the *U. simensis* leaf extract had no effect on bone ossification.

## 5. Limitation of the Study

The current study provides scientific evidence about the prenatal developmental toxicity of the *U. simensis* leaf extract in rat embryos and fetuses. However, it had some limitations. The first limitation was that the extract was administered only from 6 to 12 days of pregnancy. The second limitation was that the study used minimum acceptable number of experimental animals due to scarcity of resources to handle animals. The third limitation was that the current study did not assess postnatal effects of the *U. simensis* leaf extract. Thus, it was recommended to study the long-term effects of the *U. simensis* leaf extract in the postnatal period both on the parental rats and their offspring for better understanding of the toxicokinetic properties of *U. simensis* leaves.

## 6. Conclusions

The middle and high doses of hydroethanol leaf extract of *U. simensis* had detrimental effects on the development of rat embryos and fetuses. The embryo toxic effects of the *U. simensis* leaf extract were demonstrated by significant reduction in somite numbers and morphological scores. In addition, the embryonic developments of the caudal neural tube, otic system, olfactory system, forelimb, and hindlimb were significantly delayed in pregnant rats given the *U. simensis* leaf extract. Furthermore, the crown rump length and fetal weight were significantly decreased whereas fetal resorption was significantly increased in gravid rats given the *U. simensis* leaf extract. Therefore, pregnant women should be informed the potential toxic effects of consuming *U. simensis* leaves during pregnancy.

## Figures and Tables

**Table 1:** Phytochemical screening of *Urtica simensis* leaf with different solvents.

Secondary metabolites	Aqueous extract	Ethanol extract	Chloroform extract	Hexane extract
Phenols	−	+++	+	++
Flavonoids	−	++	−	+
Tannins	−	+	+	+
Terpenoids	−	+	−	−
Saponins	+	++	−	+
Glycosides	−	−	−	−
Steroids	++	++	++	++
Alkaloids	−	+++	−	+
Anthraquinones	−	−	−	−

*Note:* (−) = not detected, (+) = small amount, (++) = moderate amount, (+++) = high amount.

**Table 2:** Dietary intake and weight gain of pregnant rats treated with the *Urtica simensis* leaf extract.

Groups	Dietary intake/day (g)		Weight gain/dam (g)	
	Day-12	Day-20	Day-12	Day-20
Group I (250 mg/kg)	200.19 ± 1.02	201.09 ± 0.61	13.75 ± 0.23	18.21 ± 0.51
Group II (500 mg/kg)	199.48 ± 1.23	200.26 ± 1.05	13.12 ± 0.39	17.99 ± 0.46
Group III (1000 mg/kg)	197.82 ± 1.31	199.29 ± 0.71	12.64 ± 0.18	16.86 ± 0.32
Group IV (pair fed)	200.57 ± 1.04	201.52 ± 0.22	14.09 ± 0.43	18.40 ± 0.22
Group V (ad libitum)	200.73 ± 1.25	201.80 ± 0.31	14.11 ± 0.27	18.61 ± 0.19

**Table 3:** Embryonic growth indices of rat embryos treated with the *Urtica simensis* leaf extract.

Groups (doses)	Embryonic growth indices		
	Crown rump length/litter (mm)	Somite number/litter	Morphological score/litter
G-I (250 mg/kg)	3.90 ± 0.15	31.63 ± 0.22	43.98 ± 1.06
G-II (500 mg/kg)	3.88 ± 0.11	30.45 ± 0.52	43.07 ± 0.64
G-III (1000 mg/kg)	3.82 ± 0.14	29.03 ± 0.27∗	42.58 ± 1.05∗
G-IV (pair fed)	3.99 ± 0.18	31.86 ± 0.19	44.25 ± 0.77
G-V (ad libitum)	4.01 ± 0.02	32.04 ± 0.02	45.08 ± 0.05

^∗^Significant difference (*p* < 0.05) between 1000 mg/kg treated group and control groups (pair fed and ad libitum).

**Table 4:** In vivo embryonic development of rats treated with the *Urtica simensis* leaf extract.

Morphological end points	250 mg/kg (G-I)	500 mg/kg (G-II)	1000 mg/kg (G-III)	Pair fed (G-IV)	Ad libitum (G-V)
Number of embryos	110	107	106	110	109
Yolk sac circulation	3.14 ± 0.09	3.12 ± 0.07	3.08 ± 0.05	3.22 ± 0.02	3.17 ± 0.09
Flexion	2.15 ± 0.22	2.16 ± 0.09	2.14 ± 0.09	2.24 ± 0.01	2.24 ± 0.19
Heart	2.87 ± 0.52	2.87 ± 0.50	2.89 ± 0.51	2.85 ± 0.52	2.87 ± 0.52
Caudal neural tube	3.77 ± 0.02	3.51 ± 0.02∗	3.49 ± 0.01∗	3.82 ± 0.02	3.88 ± 0.01
Hind brain	2.71 ± 0.32	2.70 ± 0.30	2.69 ± 0.33	2.71 ± 0.34	2.71 ± 0.32
Mid brain	2.73 ± 0.31	2.73 ± 0.31	2.70 ± 0.29	2.75 ± 0.32	2.75 ± 0.31
Fore brain	2.98 ± 0.26	2.91 ± 0.22	2.82 ± 0.20	2.99 ± 0.27	2.99 ± 0.30
Otic system	2.54 ± 0.08	2.47 ± 0.05	2.11 ± 0.05∗	2.67 ± 0.26	2.67 ± 0.28
Optic system	2.49 ± 0.16	2.49 ± 0.13	2.40 ± 0.16	2.52 ± 0.18	2.52 ± 0.20
Olfactory system	0.87 ± 0.52	0.58 ± 0.30∗	0.53 ± 0.22∗	0.88 ± 0.53	0.89 ± 0.52
Branchial bars	2.91 ± 0.28	2.90 ± 0.27	2.89 ± 0.28	2.92 ± 0.29	2.92 ± 0.28
Maxillary process	1.37 ± 0.64	1.36 ± 0.61	1.32 ± 0.65	1.37 ± 0.63	1.38 ± 0.64
Mandibular process	0.9 ± 0.07	0.89 ± 0.04	0.82 ± 0.07	0.9 ± 0.10	0.9 ± 0.12
Fore limb	1.88 ± 0.67	1.65 ± 0.37	1.07 ± 0.39∗	1.90 ± 0.61	1.91 ± 0.57
Hind limb	1.79 ± 0.45	1.68 ± 0.05	1.09 ± 0.85∗	1.82 ± 0.75	1.89 ± 0.73

^∗^Significant difference (*p* < 0.05) as compared with control groups (pair fed and ad libitum).

**Table 5:** Fetal outcomes of pregnant rats treated with the *Urtica simensis* leaf extract.

Groups	Implantation site/dam	Resorption site/dam	Live fetuses/dam	Dead fetuses/dam
G-I (250 mg/kg)	10.28 ± 0.07	0.25 ± 0.21	10.15 ± 0.25	0.00 ± 0.00
G-II (500 mg/kg)	10.30 ± 0.09	0.32 ± 0.22	10.13 ± 0.13	0.00 ± 0.00
G-III (1000 mg/kg)	10.16 ± 0.05	0.54 ± 0.14∗	10.11 ± 0.14	0.00 ± 0.00
G-IV (pair fed)	11.08 ± 0.14	0.20 ± 0.02	11.07 ± 0.12	0.00 ± 0.00
G-V (ad libitum)	11.07 ± 0.21	0.19 ± 0.01	11.06 ± 0.13	0.00 ± 0.00

^∗^Significant difference (*p* < 0.05) as compared with control groups (pair fed and ad libitum).

**Table 6:** Fetal growth and placental weight of pregnant rats treated with the *Urtica simensis* leaf extract.

Groups	Litter weight/fetus (g)	CRL/fetus (cm)	Placental weight/fetus (g)
G-I (250 mg/kg)	3.25 ± 0.21	4.19 ± 0.15	0.52 ± 0.11
G-II (500 mg/kg)	3.22 ± 0.13	4.11 ± 0.01	0.53 ± 0.02
G-III (1000 mg/kg)	3.06 ± 0.11∗	4.07 ± 0.11∗	0.51 ± 0.18
G-IV (pair fed)	3.40 ± 0.04	4.49 ± 0.03	0.58 ± 0.12
G-V (ad libitum)	3.39 ± 0.12	4.51 ± 0.01	0.57 ± 0.15

Abbreviation: CRL = crown rump length.

^∗^Significant difference (*p* < 0.05) compared with control groups (pair fed and ad libitum).

**Table 7:** External anomalies of 20-days-old rat fetuses treated with the *Urtica simensis* leaf extract.

Groups	External anomalies							
	AC	EC	SB	KY	SC	LD	MT	AEG
Group 1 (250 mg/kg)	0	0	0	0	0	0	0	0
Group II (500 mg/kg)	0	0	0	0	0	0	0	0
Group III (1000 mg/kg)	0	0	0	0	0	0	0	0
Group IV (pair fed)	0	0	0	0	0	0	0	0
Group V (ad libitum)	0	0	0	0	0	0	0	0

Abbreviations: AC, anencephaly; AEG, external genitalia agenesis; EC, exencephaly; KY, kyphosis; LD, limb defect; MT, missed tail; SB, spina bifida; SC, scoliosis.

**Table 8:** Visceral anomalies of 20-days-old rat fetuses treated with the *Urtica simensis* leaf extract.

Groups	Visceral anomalies										
	HC	MO	AO	CP	NSD	REAA	VSD	DH	RA	HU	CT
Group 1 (250 mg/kg)	0	0	0	0	0	0	0	0	0	0	0
Group II (500 mg/kg)	0	0	0	0	0	0	0	0	0	0	0
Group III (1000 mg/kg)	0	0	0	0	0	0	0	0	0	0	0
Group IV (pair fed)	0	0	0	0	0	0	0	0	0	0	0
Group V (ad libitum)	0	0	0	0	0	0	0	0	0	0	0

Abbreviations: AO, anophthalmia; CP, cleft palate; CT, cryptorchid testes; DH, diaphragmatic hernia; HC, hydrocephalus; HU, hydroureters; MO, microphthalmia; NSD, nasal septal defect; RA, renal agenesis; REAA, retroesophageal aortic arch; VSD, ventricular septal defects.

**Table 9:** Axial bones ossification delays of 20-days-old rat fetuses treated with the *Urtica simensis* leaf extract.

Groups	Ossification delay (%)						
	HDa	SMa	RBb	CVc	TVb	LVc	SCVd
Group 1 (250 mg/kg)	0	19.5	0	0	0	0	9.4
Group II (500 mg/kg)	0	20	0	0	0	0	10.7
Group III (1000 mg/kg)	0	22	0	0	0	0	13
Group IV (pair fed)	0	17	0	0	0	0	9
Group V (ad libitum)	0	16.45	0	0	0	0	9

Abbreviations: CV, cervical vertebrae; HD, hyoid bone; LV, lumbar vertebrae; RB, ribs; SCV, sacrocaudal vertebrae; SM, sternum; TV, thoracic vertebrae.

^a^No ossification signs on the hyoid bone and less than 4 ossification centers on the sternum.

^b^No ossification signs on the ribs and less than 13 ossification centers on the thoracic vertebrae.

^c^Less than 7 ossification centers in cervical vertebrae and less than 5 ossification centers in lumbar vertebrae.

^d^Less than 4 ossification centers in sacrocaudal vertebrae.

**Table 10:** Extremity bones ossification delays of 20-days-old rat fetuses treated with the *Urtica simensis* leaf extract.

Groups	Ossification delay (%)			
	Metacarpals^a^	Metatarsals^a^	FL phalanges^b^	HL phalanges^b^
Group 1 (250 mg/kg)	6.7	6.5	5.8	6
Group II (500 mg/kg)	9	7	8	6.4
Group III (1000 mg/kg)	10	8	8.5	7
Group IV (pair fed)	7	6	6.8	5
Group V (ad libitum)	6.9	5.9	6.5	5.3

Abbreviations: FL = forelimb, HL = hindlimb.

^a^Less than 3 metacarpus and less than 3 metatarsus.

^b^No proximal phalanges of forelimb and no proximal phalanges of hindlimb.

## Data Availability

The data used to support the findings of this study are available from the corresponding author upon reasonable request.
